# A comparison of *Echium*, fish, palm, soya, and linseed oil supplementation on pork quality

**DOI:** 10.5713/ab.22.0362

**Published:** 2023-05-02

**Authors:** Barbara Elizabeth van Wyngaard, Arno Hugo, Phillip Evert Strydom, Foch-Henri de Witt, Carolina Henritta Pohl, Arnold Tapera Kanengoni

**Affiliations:** 1 Department of Animal Science, University of the Free State, Bloemfontein, 9301, South Africa; 2 Department of Animal Sciences, Faculty of Agrisciences, Stellenbosch University, Stellenbosch, 7602, South Africa; 3 Department of Microbiology and Biochemistry, University of the Free State, Bloemfontein, 9301, South Africa; 4 Johannesburg City Parks and Zoo, Johannesburg, 2000, South Africa

**Keywords:** *Echium* Oil, Lipid Stability, Omega-3 Fatty Acids, Pork Meat Quality, Sensory Analysis

## Abstract

**Objective:**

Long chain n-3 polyunsaturated fatty acids (PUFA) exert positive effects on human health. The long chain n-3 PUFA of pork can be increased by adding fish oil to the diet. Due to the cost and availability of fish oil an alternative source must be found.

**Methods:**

This study evaluated the effect of five dietary oils on meat quality, fatty acid composition and lipid stability. The five diets contained 1% palm oil (Control), 1% soya oil, 1% linseed oil, 1% fish oil, and 1% *Echium* oil, respectively. The trial consisted of 60 gilts, randomly allocated to five groups.

**Results:**

All color parameters, extractable fat content, fat free dry matter, and moisture content of the m. longissimus muscle were unaffected by dietary treatment. Consumers and a trained sensory panel could not detect a difference between the control samples and the *Echium* oil sample during sensory analysis. Samples containing higher levels of PUFA (soya, linseed, fish, and *Echium* oil) had higher levels of primary and secondary lipid oxidation products after refrigerated and frozen storage. However, these values were still well below the threshold value where off flavors can be detected. The *Echium* oil treatment had significantly higher levels of long chain PUFA than the linseed oil treatment, but it was still significantly lower than that of the fish oil treatment.

**Conclusion:**

*Echium* oil supplementation did not increase the levels of n-3 to the same extent as fish oil did. The result did however suggest that *Echium* oil can be used in pig diets to improve muscle long chain n-3 fatty acid content without any adverse effects on meat quality when compared to linseed, soya, and palm oil.

## INTRODUCTION

Since it was discovered that the Greenland Eskimos had low incidences of ischemic heart disease, despite their high fat intake, the role of omega 3 fatty acids (n-3 FA) in the diet have been investigated. It is mainly the long chain n-3 FA, eicosapentaenoic acid (EPA; C20:5n-3) and docosahexaenoic acid (DHA; C22:6n-3) shown to be beneficial to human health. Due to these benefits, it is recommended that EPA and DHA be included in human diets at an average intake of 250 to 500 mg/d [1]. Since the primary source of EPA and DHA is fish and fish oil, it is not surprisingly that most western diets fall short of the recommended intake, indicating the importance of n-3 FA supplementation. This can be achieved by either encapsulated fish oil, increasing consumption of fish and fish products high in n-3 FA or by enriching food that is familiar to the consumer, such as meat and eggs.

Pigs are the ideal animal to produce fortified meat since the fatty acid profile of meat can be changed by altering the dietary FA. Feeding fish oil to pigs is an effective method to increase the long chain n-3 polyunsaturated fatty acids (PUFA); however, due to the price of fish oil and the global decline in fish stock, this will not be sustainable. Plants containing α-linolenic acid (ALA; C18:3n-3) have been used to increase the LC n-3 PUFA of pork. Huang et al [2] reported that ALA is a precursor of LC n-3 FA and is found in linseed oil, canola oil, chia seed, camelina oil and even some nuts. The majority of studies conducted on linseed oil enriched pork found little to no increase in the DHA levels in muscle. A possible explanation for the low conversion is the initial enzyme in the PUFA biosynthetic pathway, Δ6-desaturase, which is rate-limiting. Also, ALA and linoleic acid (LA; C18:2n6) compete for the same desaturase enzymes. Therefore, DHA synthesis can be limited when the dietary ratio of LA to ALA is high. Due to this low conversion efficiency of ALA into DHA, alternatives should be investigated. Stearidonic acid (SDA; C18:4n-3) lies in a more advanced position than ALA in the PUFA biosynthetic pathway. It bypasses the Δ6-desaturase enzymes and will be more efficient than ALA in increasing the EPA and DHA n-3 in pork. A source high in SDA is *Echium plantagineum*; which is indigenous to the Western Cape province of South Africa, where it flourishes by growing abundantly in pastures. Various authors have used *Echium* oil to increase the levels of n-3 FA in fish, chicken, lamb and humans [2]. Only one study has been carried out on pork [3], but they did not investigate the effect of *Echium* oil on the n-3 deposition in meat. However, Hugo et al [4] reported that dietary supplemented *Echium* oil could be effective in altering the fatty acid profile and quality of subcutaneous fat of pork. The objective of this paper is to evaluate the effect of *Echium* oil on the fatty acid composition and meat quality of pork using the loin muscle as reference (*m. longissimus*).

## MATERIALS AND METHODS

### Animals and diet

The animals and dietary treatments were the same as reported by Hugo et al [4]. Sixty crossbred gilts (Landrace×Large White), with an average live weight of ±30 kg were used for the experiment. They had *ad libitum* access to feed and water. The experimental treatments consisted of a control diet, supplemented with 1% palm oil (BergaFat HPL-160), and four experimental diets, supplemented with 1% soya oil, 1% linseed oil, 1% fish oil, and 1% *Echium* oil. The palm oil diet had the highest levels of saturated fatty acids (SFA) and lowest levels of n-3 fatty acids and was therefore used as the control diet, while the soya oil diet had the highest total PUFA and total n-6 fatty acids (n-6 FA). While the linseed oil diet had the highest levels of total n-3 FA. LC n-3 FA such as EPA, docosapentaenoic acid (DPA), and DHA, were only recorded in the fish oil diet. The *Echium* oil diet recorded the highest levels of SDA [4]. Ethical approval for the project was given by the Agriculture Research Council Ethics Committee (APIEC14/015) and the Animal Ethics Committee of the University of the Free State (25/2014).

### Slaughter and carcass measurements

Pigs were slaughtered when the average live weight reached approximately 104 kg, as described by Hugo et al [4]. Pigs were transported by a trailer for approximately 500 m to the abattoir, where they were humanely slaughtered on arrival. All pigs were electrically stunned (220 V at 60 Hz for 7 seconds), stuck, exsanguinated, scalded (60°C), and dressed, following commercial procedures. The pH values were measured 45 minutes (pH_45 min_) as well as 24 hours (pH_24 h_) post mortem in the *M. longissimus lumborum* muscle with a portable pH-meter (Eutech instruments Pty Ltd, Singapore) with a glass body, gel-filled, spear tip probe, with Bayonet Neill-Concelman (BNC) connector.

### Tissue sampling

A core sample of *M. longissimus thoracis* was taken 24 hours post mortem, from all carcasses, 45 mm from the carcass midline between the second and third last rib, on the left side of the carcass. These samples for lipid extraction were stored in Nunc cryotubes (AEC-Amersham, Johannesburg, South Africa) at −20°C, pending lipid extraction. The loin, consisting of the *m. longissimus thoracis* and *m. longissimus lumborum* muscles, was removed from the right side of the carcass between the 12 and 13th rib. The loin was then cut into 1.5 cm thick loin chops (bone and fat attached), vacuum-packed and stored frozen at −20°C until needed for stability tests (*m. longissimus thoracis*) and sensory analysis (*m. longissimus lumborum*).

Muscle color (L*, a*, and b* values) were determined on one loin chop, after a 30 min bloom time, with a Minolta CM-600d tristimulus color analyzer, using illuminant A with specular component included. Saturation index (SI), which is related to the color intensity of the meat, was calculated according to the formula: $\text{SI}=\sqrt{a^{*2}+b^{*2}}$. Hue angle was calculated according to the formula tan^−1^ (b*/a*).

### Lipid extraction, fractionation and fatty acid analysis

The complete lipid extraction, fractionation and fatty acid analysis method was described by Hugo et al [4]. Extraction of total lipids from a 5 g muscle sample was performed using the method of Folch et al [5], using chloroform and methanol in a ratio of 2:1. The extracted lipids were converted to methyl esters by base-catalysed transesterification. Fatty acid methyl esters (FAME) were quantified, using a Varian 430-GC flame ionization GC, with a fused silica capillary column, (Chrompack CPSIL 88; 100 m length, 0.25 mm ID, 0.2 μm film thicknesses). Galaxy Chromatography Data System Software recorded the chromatograms. Identification of sample FAME was made by comparing the relative retention times of FAME peaks from samples, with those of standards obtained from Supelco (Supelco 37 Component Fame Mix 47885-U; Sigma-Aldrich Aston Manor, Pretoria, South Africa).

### Sensory analysis of pork loin chops

#### Descriptive sensory analysis of the sensory properties of fresh pork loin chops

##### i) Training of the sensory panel

A trained, 10 member sensory panel was used for the sensory analysis of the pork loin chops. The purpose of the descriptive analysis was to determine how the samples from the experimental treatments differed in terms of specific sensory characteristics. The 10 panellists were selected, based on their previous participation in descriptive sensory panels, taste and smell acuity, interest, ability to discriminate between the four basic tastes and being available for the entire study. Sample evaluations were performed in individual sensory booths, under red light conditions to mask color differences. The sensory analysis facilities conform to the American Society for Testing and Materials (ASTM) design guidelines for sensory facilities. The analyses were conducted over an 8-day period (including training). Samples from all treatments were randomly assigned to five sessions (10 min apart) per day. All samples were coded with random three-digit codes. Water, at room temperature, was served as palate cleanser in between evaluation sessions.

During the training sessions, panelists were exposed to the samples originating from the trail to be evaluated, in order to develop relevant terminology. The 10 panelists received a representative sample of pork loin meat and fat from the five dietary treatments and were then trained to increase sensitivity and ability to discriminate between specific samples and sensory attributes. In order to ensure that panelists were not influenced in any way, no information, with regard to the nature of the samples, was provided. A clear definition of each attribute was developed to describe the specific attribute to be evaluated. Panelists were instructed to give a detailed description of the aroma, flavor and aftertaste attributes of the pork loin meat and fat samples. An eight-point intensity scale was used for scoring the different characteristics of the pork loin meat and fat from the experimental treatments.

##### ii) Preparation of sensory samples

The frozen pork loin chops (stored at −20°C) were thawed over a period of 24 hours at 5°C, before cooking. The cuts were prepared and evaluated according to the American Meat Science Association and National Livestock and Meat Board (Chicago, IL, 1995) research guidelines for the cookery and sensory evaluation measurements of fresh meat. The samples were cooked, according to an oven-broiling method, using direct radiant heat. Four 1.5 cm thick loin chops with subcutaneous fat (rind on) and bone intact from a single treatment were placed on an oven pan on a rack, to allow meat juices to drain during cooking. The loin chops were positioned 90 mm below the pre-heated element of an electric oven (Mielé, H217 ovens), at an oven temperature of 170°C. As the heat radiates from only one direction, samples were turned every 10 minutes during cooking. The samples were cooked to an internal temperature of 75°C to 77°C, at the geometric center of the loin chop. Three of the hot samples were prepared immediately for sensory evaluation. The fourth loin chop was used for shear force determination. Each panelist received a standardized meat cube, measuring 12 mm ×12 mm×12 mm of each cooked sample. Only the center cubes were used and the outer sides were avoided. The meat cubes were wrapped in three-digit coded foil squares (90 mm ×90 mm) and presented at 55°C on pre-warmed plates to the panel. Panel members were instructed to bite across the grain for the determination of the first bite tenderness.

#### Consumer analysis of sensory properties of pork loin chops

##### i) Consumer sensory panel

Sensory analysis was carried out on all five treatment groups, using a 100-member untrained consumer panel, consisting of 72 females and 28 males from the Agriculture Building at the University of the Free State, with 79% of panelist between the age of 20 and 29 years. A nine-point hedonic scale, ranging from 1 for dislike extremely up to 9 for like extremely was used to score aroma, taste, aftertaste and overall acceptability as attributes.

##### ii) Preparation of sensory samples

Preparation of samples for untrained consumer sensory analysis was done as described for trained sensory panel in Section 2.5.1.2.

### Physical and chemical properties of *M. longissimus thoracis*

#### M. longissimus thoracis and backfat area measurements

The eye muscle area and backfat from the M. longissimus thoracis loin chops were traced onto transparency film. The areas were measured by means of a Video Image Analyzer (VIA) (analysis Life Science system). The composition of loin chops was expressed as % lean and % fat.

#### Drip loss of M. longissimus thoracis

Drip loss of pork loin was measured in duplicate using a revised method of Honikel [6]. Fresh meat (24 hours post-mortem) was sliced into cubes of 10 mm×10 mm×20 mm. Each cube was hung onto a pin, secured to the cap, inside a sample bottle (200 mL), ensuring that the meat did not touch the sides of the bottle. The samples were stored for 3 days at 4°C±1°C. The amount of drip loss was measured as the difference between the sample mass before and after. Drip loss was expressed as a percentage of the starting mass.

#### Water-holding capacity of M. longissimus thoracis

A 400 to 600 mg meat sample was placed on a filter paper (Whatman 4; Whatman International Limited, Maidstone, England), sandwiched between two perspex plates and pressed at constant pressure of 1,471 kPa (15 kg/cm^2^) for 5 min, according to the method described by Grau and Hamm [7]. The water holding capacity (WHC) was determined by calculating the ratio of meat area to liquid area after pressing. The areas were measured by means of a VIA (analysis Life Science system), described by Irie et al [8]. The WHC was expressed as the area of the meat, divided by the area of the moisture (including meat area).

#### Physical texture analyses

Physical texture analyses (shear force measurements) were performed with the Instron Universal Testing Machine (UTM, Model 430; Norwood, MA, USA), to compare the results regarding tenderness, with the findings of the trained sensory panel. After cooking the loin chops for sensory analysis, five loin chops, one per treatment were cooled down at room temperature for 5 to 6 hours, before shear force measurements were made. Cylindrical samples (6 cores/sample), with a 12.7 mm core diameter, were cored parallel to the grain of the meat and sheared perpendicular to the fiber direction, using a Warner Bratzler shear device mounted on the Instron Universal Testing Machine (UTM, Model 430; USA). The shear force was determined using cross head speed = 200 mm/minute and test speed with a 1 kN load cell. The reported shear force value, in Newton represents the average of the peak force measurements of each sample.

#### Myofibrillar fragment lengths

Myofibrillar fragment length (MFL) determinations were done on days 1 and 5 post mortem. A frozen *M. longissimus lumborum* sample (3 g) was placed in a 50 mL Bühler glass, containing MFL extraction buffer (30 mL) (0.02 M potassium phosphate buffer, containing 100 mM KCl, 1 mM MgCl_2_, 1 mM ethylenediaminetetraacetic acid and 1 mM NaN3 [pH 7.0] [4°C]). Samples were allowed to thaw for 60 seconds and were then homogenized for exactly 30 seconds in a Bühler HO 4/A homogenizer, at 20,000 rpm (blade turned around in order to fragment myofibrils rather than cut them). Samples were subsequently centrifuged at 3,000 rpm (4°C) for 15 min. The supernatant was discarded, and the pellet was suspended in MFL extraction buffer (30 mL) (4°C) and centrifuged at 3,000 rpm (4°C) for 15 min. The supernatant was once again discarded and the pellet suspended in MFL extraction buffer (10 mL) (4°C). The suspension was filtered under vacuum through a 1,000 μm polyethylene strainer. Additional MFL extraction buffer (5 mL) (4°C) was used to facilitate the passing of myofibrils through the strainer. The filtrate was subsequently filtered under vacuum, through a 250 μm polyethylene strainer. This filtrate was used to measure MFL with the VIA. Myofibrils were extracted, according to Culler et al [9], with some modifications. The myofibrillar fragments from the filtrate were examined with an Olympus BX40 system microscope at a 400× magnification. One hundred myofibril fragments of each sample were measured, using analysis Life Science software package.

### Chemical and oxidative stability studies

#### Lipid stability of fresh and frozen pork loin chops

Four loin chops from each pig, from each treatment group, were individually packed. One loin chop from each pig was packed into a polystyrene tray, containing an absorbent pad and overwrapped with oxygen-permeable polyvinyl chloride meat stretch wrap, and stored at 4°C for 7 days, under fluorescent light, for the fresh meat stability study. The remaining three loin chops were vacuum packed. Of these, one loin chop was used for lipid stability tests at day 0 and the other two stored at −18°C, in the dark, for frozen storage stability studies at 3 and 6 months.

To assess oxidative stability during frozen storage, lean meat and backfat were sampled on day 0, as well as at 3 and 6 months. Thiobarbituric acid reactive substances (TBARS) analyses, for determination of lipid oxidation of lean meat, were done according to the following method. Two 5 g samples lean meat were removed from the middle of each loin chop and the aqueous acid extraction method of Raharjo et al [10] was used. A 10 g sample of backfat (inner + outer layer) was also removed for lipid extraction, using the Folch et al [5] method. To assess lipid oxidation in backfat, the PV was determined on 5 g of the extracted lipid sample, using the AOAC [11] method nr. 965.33.

### Statistical analysis

One-way analysis of variance (ANOVA) was performed to determine significant differences between the chemical, physical, histological, chemical stability and sensory properties of meat from the five dietary treatments [12]. A two-way ANOVA was performed to determine the effect of time on MFL, Peroxide values and TBARS [12]. The Tukey-Kramer multiple comparison test (α = 0.05) was carried out to identify significant differences between the treatment means [12]. In tables, statistically analyzed data are represented with ±standard deviations.

## RESULTS

### Physical and chemical properties of *M. longissimus thoracis*

There was no significant difference between dietary treatments for loin chop composition such as eye muscle area, fat area of the chop, meat area of the chop, fat area (%) or meat area (%) (Table 1). None of the color measurements (L*-, a*-, b*-value, chroma, and hue angle) or extractable fat content were significantly influenced by dietary oil treatments (Table 1). The pH_45_ and temperature_45_ of the treatments differed significantly 45 minutes post-mortem (Table 1). The *Echium* oil treatment had a higher (p = 0.029) pH (6.50) compared to the soya oil treatment (6.22) and a lower (p< 0.001) temperature (36.08°C) than the control (38.03°C) treatment. It is interesting to note that despite differences recorded at 45 minutes post-mortem, there were no differences (p> 0.05) between treatments for either pH of temperature at twenty-four hours post-mortem. From Table 1, it is further evident that the *Echium* oil treatment had less (p = 0.049) drip loss (8.18%) than the control (10.79%) and fish oil treatments (9.42%), while the WHC of the linseed oil treatment (0.36) were the highest (p = 0.021). The control treatment had a significantly lower iodine value (68.71) than the fish oil (76.06) treatment.

### Fatty acid composition of intramuscular fat from *M. longissimus thoracis*

Despite differences (p<0.05) in individual fatty acid profiles between treatments, there were no significant differences between treatment means in terms of intramuscular fat (IMF) for both the total saturated fatty acids (∑SFA) and monounsaturated fatty acids (∑MUFA) as seen in Table 2. Even though statistically significant (p<0.001), the levels of SDA of the *Echium* oil treatment (0.03%) were only slightly higher than the other treatments (0.01%) (Table 2). The linseed oil treatment had the highest (p = 0.047) levels of total PUFA (∑PUFA) and there was no significant difference between the control, *Echium* and fish oil treatments. The fish oil treatment had a higher (p<0.001) total n-3 FA as well as total LC n-3 FA than all other treatments. This resulted in the fish oil treatment having the most favorable n-6/n-3 ratio followed by the *Echium* and linseed oil treatments. The ratio of PUFA to SFA did not differ (p = 0.063) between treatments (Table 2).

Table 3 represents the actual n-3 FA expressed as mg/100 g muscle. From this table it is evident that the actual SDA content (0.59 mg/100 g) of the *Echium* oil treatment, was higher (p<0.001) than of the other treatments (0.01 mg/100 g). The *Echium* oil treatment also resulted in higher (p<0.001) actual levels of EPA (2.44 mg/g) and DPA (7.87 mg/g) compared to the linseed oil (2.33 mg/g and 5.34 mg/g) treatment in IMF tissue. The fish oil treatment still had significantly higher levels (p<0.001) of EPA and DPA than all other treatments. The levels of DHA were highest in the fish oil treatment. There was no significant difference between the fish oil and *Echium* oil treatments for the total n-3 fatty acids, but the sum of the EPA, DPA, and DHA was higher (p<0.001) in the fish oil treatment (Table 3).

### Physical characteristics of *M. longissimus lumborum*

Dietary lipid treatment had no effect (p>0.05) on physical characteristics such as cooking loss, cooking drip loss, evaporation loss, thawing loss, shear force measurements (Table 4) or on MFL (Table 5).

### Sensory analysis of pork *M. longissimus lumborum*

#### Descriptive sensory analysis

While the trained panel did detect (p<0.05) a fishy aroma, flavor and after taste in the fish oil sample (Table 6). It is further interesting to note that the fish oil treatment recorded the lowest (p<0.05) aroma for fresh cooked pork meat (4.87) which was linked with the highest (p<0.05) values for rancid/old meat aroma (1.46) and meat flavor (1.52). The *Echium* oil treatment did not differ significantly from the control treatment for meat aroma, flavor and aftertaste.

#### Consumer sensory analysis

There was no significant difference between treatments for aroma (Table 7). The control ranked significantly higher for taste, aftertaste and overall acceptability than the fish oil treatment (Table 7).

### Lipid stability of fresh and frozen pork loin chops

Already on day 0, the soya, linseed, fish and *Echium* oil treatments had higher PV values (Table 8). The same was seen on day 7. After 3 months frozen storage the linseed, fish and *Echium* oil treatments, which also had the higher levels of LC n-3 PUFA, recorded significantly higher PV values than the control. After 6 months of frozen storage there were no significant differences between treatments (Table 8).

Diet lipid treatment had no influence on the TBARS values at day 0 (Table 9). After 7 days refrigerated storage, the treatments containing PUFA did record higher (p = 0.010) TBARS values. The same tendency was seen after 3 and 6 months frozen storage (Table 9).

## DISCUSSION

### Physical and chemical properties of *M. longissimus thoracis*

There was no significant difference between dietary treatments for loin eye muscle area, fat area of the chop, meat area of the chop, fat area (%), or meat area (%) (Table 1).

The pH and temperature early post mortem affects the quality of pork and can have an influence on the color, texture, WHC, drip and cooking loss [13]. Pork can be classified as red, firm and non-exudative (RFN), pale, soft and exudative (PSE) or dark, firm and dry (DFD). Normal meat has a pH_24 h_ in the order of 5.5. Meat with a pH_24 h_ of >6.0 is classified as DFD [13]. It is evident from Table 1 that none of the treatment groups could be classified as DFD and there were no significant differences between treatments for pH_24 h_. A sudden drop in pH at a high temperature result in PSE meat [13]. PSE meat is characterized as having a pale color, soft texture, high drip loss and low WHC [13]. Pork with a pH_45 min_ of <5.7 to 6.0 can be classified as PSE pork.

Water holding capacity is the ability of meat to retain water under external influences such as compression or centrifugation. Therefore, higher values for WHC mean that less moisture was pressed out of the products. Water holding capacity is important to the consumer as it influence the tenderness, juiciness, firmness and appearance of meat. A higher WHC will increase the value of pork for use in highly processed pork products. Although statistically significant (p = 0.0021), WHC did not show much variation among treatments (Table 1). The WHC of the linseed oil treatment was significantly higher than the control.

Meat color is regarded as a visual measure of freshness and quality that influences the choice of consumers at the point of sale. Although these objective/instrumental measurements of color do not by itself reflect consumer acceptance, studies have shown that thresholds for instrumental color parameters can be used to estimate consumer acceptance for beef and lamb. However, in pork it seems consumers are not as strongly influenced by color. Studies showed that consumers prefer a red color either pale or dark [14]. None of the color measurements (L*, a*, b*, chroma, or hue) were influenced by diet.

Intramuscular fat is important in terms of the sensory quality of pork and has been positively related to flavor, juiciness and tenderness. De Vol et al [15] suggested that pork should have an IMF of 2% to 3% to ensure desirable palatability. Only the control and *Echium* oil treatments had IMF content of above 2% (Table 1), however there were no significant difference for extractable fat between treatments. Iodine value differed significantly (p = 0.025) between treatments with the control treatment having an IV of less than 70, as recommended by Barton-Gade [16].

### Fatty acid composition of IMF from *M. longissimus thoracis*

The quantitative FAME estimation (Table 2) showed no difference (p>0.05) between the linseed oil and *Echium* oil treatments for EPA and DPA, although the actual amounts (mg/100 g muscle) showed that the *Echium* oil treatment had higher (p<0.001) levels of both EPA and DPA than the linseed treatment (Table 3). The actual levels of EPA, DPA, and DHA were the highest in meat from the fish oil treatment (Table 3). Adding *Echium* oil to the diets of pigs caused in increase in both the EPA and DPA content of meat. While the *Echium* oil treatment had higher (p<0.001) actual levels of DHA when compared to the control and soya oil treatment, it did not differ (p<0.001) from the linseed oil treatment (Table 3). These findings agree with various authors who found that the consumption of Echium and linseed oil increased the EPA and DPA in human plasma, but had little effect on the DHA levels [17].

The enrichment achieved in terms of the SDA and EPA may be of some health benefit to consumers. The Japanese Lipid Study [18] demonstrated that long-term supplementation of pure EPA, in the absence of supplemental DHA, significantly reduced major coronary events in patients with a history of cardiovascular heart diseases. In a comparison experiment Harris [19] evaluated the impact of supplemental ALA, SDA, and EPA on changes to the omega-3 index. The omega-3 index is a clinical biomarker for cardiovascular disease and correlates well with the risk for various cardiovascular disease endpoints. The omega-3 index is calculated as the sum of EPA and DHA in erythrocyte membranes expressed as a percentage of total erythrocyte fatty acids. Harris [19] found that SDA and EPA were more effective than ALA (which had no effect) in improving the omega-3 index and that SDA was 17% as effective as EPA.

The label claim categories for ‘source’ and ‘good source’ reflect the degree to which a meal contributes to the consumer achieving daily values for a nutrient. Food Standards Australia and New Zealand’s cut off points for ‘source’ and ‘good source’ for n-3 LC-PUFA are 30 and 60 mg/serving, respectively [20]. By considering these criteria it is possible to categorize the meat from the *Echium*, fish and linseed oil treatments as a source of total n-3 FA (Table 3), while the pork meat from the fish oil treatment could additionally be labelled as a source of EPA+DPA+DHA (Table 3). Under the US Food and Drug Administration guidelines meals which provide 10% to 19% of the recommended daily intake can be labelled as a ‘good source’, while those which provide 20% or more can be labelled ‘high’, ‘rich in’, or ‘excellent source’ [21].

To promote human health, a relatively low n-6:n-3 ratio, from an adequate intake of n-3 fatty acids is recommended. Scollan et al [22] recommended that the n-6:n-3 PUFA ratio be limited to 4:1. The fish oil treatment had the highest total n-3 FA and therefore the most favorable n-6:n-3 ratio. Ulbricht and Southgate [23] suggested that the ratio of PUFAs to SFAs (P:S) should be at least 0.4. The soya oil, linseed oil and fish oil treatments all had P:S of more than 0.4 (Table 2).

### Physical characteristics of *M. longissimus lumborum*

Cooking loss is relevant to the consumer as it determines the final yield of the cooked product and affects the juiciness and tenderness perceptions. According to various consumer studies, drip loss is one of the most relevant intrinsic cues for consumers to determine pork quality. However, Verbeke et al [24] found that drip loss is not perceived as a major hurdle for the acceptance of pork loin chops as it is overlooked due to the presence of other more desirable/undesirable attributes. Results in Table 4 indicate that there were no significant differences between dietary treatments in terms of cooking, cooking drip, evaporation or thawing losses. These findings are in agreement with the results of Miller et al [25] as well as that of Scheeder et al [26].

It further seems that dietary oil supplementation did not affect the shear force measurements as illustrated in Table 4. The lack of differences in terms of the extractable fat in muscle between treatments as seen in Table 1, may partly explains the similar shear force obtained for these treatments. Current results are in agreement with that of Miller et al [25] as well as Scheeder et al [26] who also did not find any effect on shear force when pigs were fed diets containing safflower oil, sunflower oil, canola oil, olive oil and soyabean oil.

During the aging of meat, the myofibrillar structure is broken down by calpain (a proteolytic enzyme), therefore, shorter MFL are normally associated with a higher degree of proteolysis and supposedly a larger degree of ageing, and, therefore, tenderization. In the present study it was evident that there were no significant differences between treatments for MFL on either day 1 or 5 post mortem (Table 5). This corresponds with the lack of differences among treatments for shear force tenderness.

### Sensory analysis of pork *M. longissimus lumborum*

#### Descriptive sensory analysis

The aroma of the meat from the fish oil treatment had a fishy aroma (p<0.001) and less of a fresh cooked pork meat aroma (p = 0.011; Table 6). Furthermore, the fish oil treatment had higher (p = 0.049) scores for a rancid/old aroma, when compared to the control treatment (1.46 vs 1.24). The fish oil treatment recorded the highest fishy taste (p<0.001) and aftertaste (p<0.001), which agrees with the findings of Wood and co-workers [27]. Although the *Echium* and linseed oil treatments had higher scores for a rancid/old taste than the control and soya oil treatments, it did not differ statistically (p>0.05). On the other hand, the fish oil treatment had higher (p = 0.010) scores for rancid/old taste, when compared to the control and soya oil treatments (Table 6). This was due to the formation of oxidation products in these treatments, with higher levels of LC PUFA. The fish oil treatment recorded lower (p = 0.023) scores for a cooked pork meat aftertaste, when compared to the control and soya oil treatments (Table 6).

#### Consumer sensory analysis of pork M. longissimus lumborum

Consumers scored the cooked pork on attributes such as aroma, taste, aftertaste and overall acceptability (Table 7), and were unable to detect a difference in aroma (p = 0.549) between the five treatments (Table 7). Feeding pork diets containing fish oil often results in meat with a fishy taste and odor, especially after long storage. Øverland et al [28] found off flavors when pigs were fed 1% to 3% fish oil. The consumer panel preferred the taste of the control treatment (6.17) and scored the fish oil treatment the lowest (5.27) (p = 0.011) for taste. The same trend was seen for aftertaste with the control (5.97) scoring the highest (p = 0.026) and fish oil (5.18) treatment the lowest. For overall acceptability the control (6.16) had significantly higher (p = 0.022) scores than the fish oil (5.38) treatment. Studying the results in Tables 6 and 7, it is interesting to note that there was not clearly defined consumer preference for pork meat from different dietary treatments, except the unacceptable fishy aroma, flavor and after taste (Table 7).

### Lipid stability of fresh and frozen pork

It is well known that higher levels of PUFA result in accelerated oxidation. On both day 0 and after 7 days of refrigerated storage, the control samples had lower peroxide (p<0.001) values (PV) than all the other treatments (Table 8). A possible explanation for the lower PV recorded by the control treatments would be that the control sample contained lower levels of unsaturated fatty acids (UFA). After 3 months of frozen storage the control treatment still had the lowest PV (p<0.001) however, it did not differ (p>0.001) from the soya oil treatment. At 6 months of frozen storage there were no significant differences between treatments (Table 8). As the secondary oxidation products form, the primary oxidation products decrease resulting in lower PV. This could possibly explain why there were no significant differences between treatments after 6 months storage. Additionally, the increase in variation as recorded by the standard deviation further illustrated that the PV at 6 months of storage is not a good indication for oxidation and it is therefore important to also test for the secondary oxidation products (TBARS).

The TBARS values presented in Table 9 were used to test for secondary oxidation products (malonaldehyde). It is clear that there were no significant differences between treatments for TBARS values at day 0. After 7 days of refrigerated storage, there were small differences (p = 0.010) between the control (0.05 mg malonaldehyde/kg meat) and the linseed oil (0.09 mg malonaldehyde/kg meat) treatment. The control still had the lowest TBARS values after 3 months of frozen storage while *Echium* oil recorded the highest value (p<0.001) (Table 9). This agrees with the findings of Bryhni et al [29] who found that after 1-month frozen storage, meat from pigs on higher PUFA diets had higher TBARS values. After another 3 months of frozen storage the TBARS values of the control treatment remained unchanged, and all other treatments increased slightly (Table 9). The linseed oil and *Echium* oil treatments had the highest values followed by the fish- and soya oil treatments. Threshold values for TBARS, where off-flavors were noted by taste panels, are rare and inconclusive, but Tarladgis et al [30] reported that off-flavors were first detected between TBARS values of 0.5 and 1.0 mg malonaldehyde/kg meat. Given the results in Table 9, it is clear that all values of the present study fall far below this threshold.

## CONCLUSION

Feeding pigs a diet containing 1% *Echium* oil had no adverse effects on meat quality. Adding 1% *Echium* and 1% linseed oil to the diets resulted in similar fatty acid profiles, however the *Echium* treatment had significantly higher levels of SDA, EPA, DPA, and total n-3 fatty acids. Due to the higher levels of n-3 fatty acids, the Echium treatment resulted in a more favorable n-6:n-3 ratio. The higher levels of PUFA caused an increase in lipid oxidation products when meat was stored under fluorescent lighting at 4°C (retail refrigeration conditions), however, these values were still far below the threshold values where consumers are able to detect off and rancid flavors. Both consumers and a trained panel were unable to detect a difference between the control and the *Echium* oil treatment during sensory analysis. The fish oil treatment had the highest levels of EPA, DPA, and DHA. This study does confirm that *Echium* oil can be used to increase the levels of LC n-3 PUFA in pork. More research is needed to evaluate whether feeding pigs different dietary supplemental levels of *Echium* oil might further increase the levels of LC n-3 PUFA in pork muscle.

## Figures and Tables

**Table 1:** Physical and quality characteristics of *M. longissimus thoracis* from the different experimental treatments (n = 10)

Items	Control	Soya oil	Linseed oil	Fish oil	Echium oil	p-value
Eye muscle area (mm^2^)	4,159±517	4,031±468	4,235±566	3,918±575	3,997±359	0.634
Fat area of chop fat (mm^2^)	5,161±925	5,292±1,289	4,052±756	4,631±1,258	4,483±568	0.058
Meat area of chop (mm^2^)	9,965±1,118	10,743±1,331	9,560±820	10,180±1,249	9,457±1,288	0.121
Fat area %	34.11±4.44	32.69±5.54	29.75±4.11	31.14±6.76	32.33±4.76	0.419
Meat area %	65.89±4.44	67.31±5.54	70.25±4.11	68.86±6.76	67.67±4.76	0.419
pH_45 min_	6.36^ab^±0.19	6.22^a^±0.28	6.41^ab^±0.25	6.23^ab^±0.19	6.50^b^±0.16	0.029
Temp_45 min_ °C	38.03^b^±1.25	36.47^ab^±0.96	35.39^a^±1.87	36.51^ab^±0.94	36.08^a^±1.28	<0.001
pH_24 h_	5.41±0.05	5.41±0.04	5.43±0.03	5.39±0.05	5.43±0.05	0.081
Temp_24 h_ °C	3.36±0.56	3.01±0.56	3.38±0.43	3.25±0.69	3.24±0.49	0.325
Drip loss (%)	10.79^b^±1.95	9.23^ab^±1.53	9.42^ab^±2.31	10.94^b^±2.43	8.18^a^±2.88	0.049
Water holding capacity (meat area/total area)	0.32^a^±0.03	0.32^ab^±0.03	0.36^b^±0.04	0.35^ab^±0.03	0.32^ab^±0.02	0.021
Colour L* - value	55.59±3.66	53.78±2.91	52.17±3.65	53.32±4.43	53.49±3.88	0.372
Color a* - value	12.24±2.43	11.09±1.74	9.53±1.87	10.58±2.62	11.20±2.96	0.159
Color b* - value	12.66±2.71	10.95±2.00	9.46±2.38	10.68±2.48	10.87±2.99	0.107
Chroma	17.62±3.61	15.60±2.60	13.46±2.92	15.07±3.53	15.63±4.16	0.125
Hue angle	45.82±1.68	44.44±2.18	44.30±3.71	45.28±2.69	44.00±2.58	0.522
Extractable fat (%)	2.05±0.40	1.90±0.49	1.86±0.43	1.82±0.39	2.14±0.34	0.397
Iodine value (calculated)	68.71^a^±4.14	73.68^ab^±5.36	74.90^ab^±6.92	76.06^b^±5.52	71.21^ab^±4.18	0.025

a,b Means with different superscripts in the same row differ significantly (p<0.05).

**Table 2:** Fatty acid composition and fatty acid ratios of *M. longissimus thoracis* of pigs from the different experimental treatments (n = 10)

Fatty acid (%)	Control	Soya oil	Linseed oil	Fish oil	Echium oil	p-value
C14:0	1.20±0.20	1.16±0.26	1.08±0.16	1.19±0.12	1.22±0.11	0.465
C16:0	25.16±1.10	24.56±1.31	24.17±0.93	24.82±1.00	25.16±0.65	0.165
C18:0	11.31±0.92	11.34±0.92	11.45±0.73	11.74±0.86	11.38±1.06	0.827
∑SFA	38.09±1.85	37.47±1.40	37.08±1.51	38.10±1.30	38.17±1.56	0.419
C16:1c9	3.31±0.37	3.04±0.65	2.88±0.33	2.95±0.50	3.08±0.27	0.277
C18:1c9	38.06±1.72	35.68±2.00	36.33±3.18	35.69±2.50	37.23±1.78	0.108
∑MUFA	48.85±1.70	45.81±2.76	46.24±3.73	45.82±3.02	47.74±1.93	0.059
C18:2c9,12 (n-6)	9.89^ab^±1.51	12.61^b^±2.64	11.71^ab^±2.97	10.94^ab^±2.17	9.61^a^±1.50	0.023
C18:3c9,12,15 (n-3)	0.75^ab^±0.09	0.85^b^±0.08	1.57^d^±0.15	0.70^a^±0.07	1.35^c^±0.12	<0.001
C18:4c6,9,12,15(n-3)	0.01^a^±0.01	0.01^a^±0.01	0.01^a^±0.01	0.01^a^±0.01	0.03^b^±0.02	<0.001
C20:5c5,8,11,14,17 (n-3)	0.04^a^±0.02	0.08^ab^±0.03	0.29^bc^±0.15	1.24^d^±0.38	0.33^c^±0.12	<0.001
C22:6c4,7,10,13,16,19 (n-3)	0.03^a^±0.03	0.04^a^±0.03	0.07^a^±0.05	0.39^b^±0.11	0.07^a^±0.04	<0.001
C22:5c7,10,13,16,19 (n-3)	0.13^a^±0.05	0.22^a^±0.07	0.44^b^±0.21	0.84^c^±0.19	0.47^b^±0.14	<0.001
PUFA (%)	13.06^a^±2.17	16.72^b^±3.54	16.68^b^±4.39	16.08^ab^±3.27	14.09^a^±2.29	0.047
n-6 (%)	12.08^ab^±2.13	15.48^b^±3.49	14.16^ab^±3.96	12.89^ab^±2.70	11.69^a^±1.98	0.038
n-3 (%)	0.98^a^±0.10	1.24^a^±0.08	2.52^b^±0.46	3.19^c^±0.63	2.37^b^±0.32	<0.001
Long chain n-3	0.20^a^±0.10	0.33^ab^±0.12	0.80^bc^±0.38	2.46^d^±0.66	0.87^c^±0.29	<0.001
PUFA/SFA	0.35±0.07	0.45±0.11	0.45±0.13	0.42±0.09	0.37±0.07	0.063
*n*-6/*n*-3	12.37^b^±2.11	12.43^b^±2.51	5.55^a^±0.70	4.05^a^±0.41	4.93^a^±0.30	<0.001

SFA, saturated fatty acids; MUFA, monounsaturated fatty acids; PUFA, polyunsaturated fatty acids.

a–d Means with different superscripts in the same row differ significantly (p<0.05).

**Table 3:** Omega-3 fatty acid content of *M. longissimus thoracis* of pigs from the different experimental treatments (mg/100 g muscle; n = 10)

Items	Control	Soya oil	Linseed oil	Fish oil	*Echium* oil	p-value
C18:3c9,12,15	12.46^a^±4.66	12.24^a^±5.03	22.15^b^±8.00	9.53^a^±3.39	23.62^b^±5.65	<0.001
C18:4c6,9,12,15	0.01^a^±0.01	0.01^a^±0.01	0.01^a^±0.01	0.01^a^±0.01	0.59^b^±0.44	<0.001
C20:3c11,14,17	0.50^a^±0.33	0.81^a^±0.46	2.33^b^±1.04	0.41^d^±0.29	2.44^c^±0.55	<0.001
C20:5c5,8,11,14,17	0.64^a^±0.25	0.96^a^±0.19	3.50^b^±0.61	15.47^d^±2.21	5.47^c^±1.50	<0.001
C22:5c7,10,13,16,19	1.94^a^±0.43	2.74^a^±0.34	5.34^b^±0.80	10.67^d^±1.58	7.87^c^±1.37	<0.001
C22:6c4,7,10,13,16,19	0.33^a^±0.39	0.50^ab^±0.33	0.91^bc^±0.45	4.86^d^±0.85	1.06^c^±0.56	<0.001
Total n-3 fatty acids	15.87^a^±4.43	17.25^a^±5.17	34.23^b^±8.84	40.94^c^±6.39	41.05^c^±7.14	<0.001
EPA+DPA+DHA	2.91^a^±0.98	4.20^a^±0.71	9.75^b^±1.59	31.00^d^±3.97	14.40^c^±3.25	<0.001

EPA, eicosapentaenoic acid; DPA, docosapentaenoic acid; DHA, docosahexaenoic acid.

a–d Means with different superscripts in the same row differ significantly (p<0.05).

**Table 4:** Physical characteristics and cooking effects on *M longissimus lumborum* from the experimental treatment groups (n = 10)

Items	Control	Soya oil	Linseed oil	Fish oil	*Echium* oil	p-value
Total cooking loss (%)	24.11±3.27	21.47±2.85	21.79±2.64	22.17±0.95	20.62±2.90	0.065
Cooking drip loss (%)	8.40±1.29	7.59±1.93	6.53±1.47	7.05±1.57	7.01±1.72	0.117
Evaporation loss (%)	15.71±2.80	13.88±2.41	15.26±2.04	16.25±2.08	13.61±2.07	0.055
Thawing loss (%)	1.78±1.02	2.09±0.80	2.04±0.83	2.23±0.72	2.37±0.88	0.617
Shear force measurement (N)	43.25±7.55	49.62±10.59	54.72±11.87	49.82±6.77	48.74±8.92	0.123

**Table 5:** Effect of dietary treatment on myofibrillar fragment lengths of *M. longissimus lumborum* of pigs (n = 10)

Items	Control	Soya	Linseed	Fish	*Echium*	p-value
Day 1 post mortem (μm)	56.16^B^±3.87	56.20^B^±4.37	56.00^B^±7.83	57.20^B^±4.12	53.60^B^±9.61	0.783
Day 5 post mortem (μm)	46.24^A^±8.65	45.76^A^±11.35	43.17^A^±13.34	47.84^A^±4.18	41.20^A^±7.53	0.554
p-value	0.004	0.014	0.015	<0.001	0.005	

Interaction between time×treatment was not significant (p = 0.9487).

A,B Means with different capital superscripts in the same column differ significantly (p<0.05).

**Table 6:** Descriptive sensory analysis of pork *M. longissimus lumborum* (pork loin) and fat samples of gilts from the experimental groups (n = 10)

Items	Control	Soya oil	Linseed oil	Fish oil	*Echium* oil	p-value
Meat aroma						
Roast pork meat	3.33±1.03	3.30±0.96	3.45±0.99	3.27±0.93	3.26±0.85	0.629
Fresh cooked pork meat	5.22^b^±0.91	5.23^b^±0.85	5.17^ab^±0.83	4.87^a^±0.85	5.25^b^±0.93	0.011
Rancid/old	1.24^a^±0.51	1.26^ab^±0.58	1.34^ab^±0.64	1.46^b^±0.69	1.27^ab^±0.45	0.049
Sour	1.75±0.64	1.77±0.55	1.72±0.62	1.81±0.56	1.74±0.60	0.857
Fishy	1.02^a^±0.14	1.05^a^±0.22	1.07^a^±0.26	1.29^b^±0.54	1.07^a^±0.29	<0.001
Piggy (old-musty)	1.37±0.61	1.31±0.53	1.34±0.55	1.43±0.67	1.33±0.55	0.637
Livery (metallic/bloody)	1.51±0.58	1.49±0.66	1.60±0.60	1.63±0.60	1.54±0.59	0.411
Meat flavor						
Roast pork meat	3.42±1.02	3.33±0.99	3.42±1.11	3.16±0.93	3.38±1.00	0.334
Cooked pork meat	5.36±0.81	5.44±0.87	5.26±0.87	5.12±0.88	5.31±0.84	0.099
Rancid/old	1.23^a^±0.42	1.27^a^±0.57	1.35^ab^±0.70	1.52^b^±0.72	1.35^ab^±0.59	0.010
Livery (Metallic/bloody)	1.87±0.65	1.85±0.69	1.91±0.70	1.97±0.64	1.99±0.76	0.534
Sour	2.43±0.62	2.25±0.72	2.37±0.75	2.44±0.72	2.28±0.71	0.201
Fishy	1.04^a^±0.20	1.07^a^±0.29	1.08^a^±0.27	1.57^b^±0.71	1.04^a^±1.20	<0.001
Piggy (old-musty)	1.34±0.54	1.36±0.63	1.43±0.59	1.52±0.67	1.41±0.59	0.247
Cooked pork meat	5.04^b^±0.89	4.99^b^±0.87	4.82^ab^±0.95	4.58^a^±0.85	4.92^ab^±0.90	0.003
After taste						
Rancid/old	1.23±0.49	1.29±0.66	1.32±0.63	1.39±0.65	1.26±0.50	0.367
Sour	2.11±0.67	1.99±0.61	2.01±0.69	2.16±0.66	1.91±0.67	0.061
Fishy	1.04^a^±0.20	1.03^a^±0.17	1.05^a^±0.22	1.45^b^±0.64	1.05^a^±0.26	<0.001
Metallic	1.84±0.58	1.83±0.60	1.86±0.65	1.87±0.61	1.92±0.56	0.853

a,b Means with different superscripts in the same row differ significantly (p<0.05).

**Table 7:** Consumer sensory characteristics analysis of *M. longissimus lumborum* (pork loin) of gilts from the experimental treatment groups (n = 10)

Items	Control	Soya oil	Linseed oil	Fish oil	*Echium* oil	p-value
Aroma	5.85±1.49	5.79±1.66	5.63±1.72	5.50±1.78	5.59±1.60	0.549
Taste	6.17^b^±1.68	5.89^ab^±1.92	5.56^ab^±2.02	5.27^a^±1.98	5.57^ab^±1.88	0.011
Aftertaste	5.97^b^±1.55	5.74^ab^±1.76	5.54^ab^±1.82	5.18^a^±1.88	5.57^ab^±1.68	0.026
Overall acceptability	6.16^b^±1.52	6.00^ab^±1.81	5.71^ab^±1.83	5.38^a^±1.98	5.84^ab^±1.60	0.022

a,b Means with different superscripts in the same row differ significantly (p<0.05).

**Table 8:** Lipid stability of subcutaneous fat from fresh pork loin chops from the experimental treatment groups stored at 4°C for 7 days and at −18°C for 3 and 6 months peroxide value (meq. peroxide/1,000 g fat) (n = 10)

Items	Control	Soya oil	Linseed oil	Fish oil	*Echium* oil	p-value
Day 0	4.84^a,A^±1.75	9.51^b,A^±3.05	12.30^b,A^±3.59	10.51^b,A^±2.45	10.33^b,A^±2.65	<0.001
Day 7	10.49^a,A^±5.12	17.76^b,AB^±5.76	21.14^b,AB^±5.54	20.40^b,A^±5.50	17.58^b,A^±3.39	<0.001
Month 3	6.37^a,A^±3.19	9.04^ab,A^±2.29	13.82^c,A^±5.03	13.65^bc,A^±3.78	11.10^bc,A^±3.49	<0.001
Month 6	17.30^B^±9.38	24.33^B^±15.77	34.39^B^±16.88	31.92^B^±15.47	29.34^B^±12.77	0.078
p-value	<0.001	0.003	<0.001	<0.01	<0.001	

Interaction of treatment on storage time was not significant (p = 0.8125).

a–c Means with different superscripts in the same row differ significantly (p<0.05).

A,B Means with different capital superscripts in the same column differ significantly (p<0.05).

**Table 9:** Lipid stability of muscle from fresh pork loin chops from the experimental treatment groups stored at 4°C for 7 days and at −18°C for 3 and 6 months1)

Items	Control	Soya oil	Linseed oil	Fish oil	*Echium* oil	p-value
Day 0	0.04^A^±0.01	0.04±0.02	0.05^A^±0.02	0.05^A^±0.02	0.05^A^±0.02	0.563
Day 7	0.07^a,B^±0.02	0.07^ab^±0.02	0.09^b,B^±0.01	0.08^ab,B^±0.01	0.09^ab,B^±0.02	0.010
Month 3	0.05^a,AB^±0.02	0.07^ab^±0.02	0.09^bc,AB^±0.02	0.09^bc,B^±0.02	0.10^c,BC^±0.02	<0.001
Month 6	0.05^a,AB^±0.02	0.08^ab^±0.06	0.12^b,B^±0.06	0.09^ab,B^±0.01	0.12^b,C^±0.04	0.002
p-value	0.006	0.162	<0.001	<0.001	<0.001	

1) TBARS value (mg malonaldehyde/kg meat) (n = 10).

Interaction of treatment on storage time was not significant (p = 0.0864).

a–c Means with different superscripts in the same row differ significantly (p<0.05).

A–C Means with different capital superscripts in the same column differ significantly (p<0.05).
